# Socio-ecological drivers of vulnerabilities of children living within orphan homes and the implications for their nurturance care

**DOI:** 10.3389/fpubh.2023.1203510

**Published:** 2023-12-12

**Authors:** Olayinka M. Onayemi, Given Hapunda

**Affiliations:** ^1^Department of Sociology, Bowen University, Iwo, Nigeria; ^2^Department of Psychology, University of Zambia, Lusaka, Zambia

**Keywords:** child protection (policy and practice), child vulnerability, child welfare, nurturing care, orphan home, Nigeria, SDG 16

## Abstract

At the heart of the Sustainable Development Goals (SDG) is the vision to “leave no one behind, and to see that all children survive, thrive and transform. However, some categories of children may remain left behind owing to their disproportionate exposure to the risk of threats and deficit of attention to the social and ecological climate that characterizes the various systems in which they are found. This study is concerned with one major question: Despite diverse local and international instruments that favor full nurturance and development of children, what social forces play as threat to full nurturance care in the context of children living in Orphan homes? Nurturing care framework and Brofenbrener’s ecological system theory were adopted as the analytical frameworks. Research design was exploratory. Data were collected through sessions of in-depth-interview with orphanage managers, caregivers, and social workers on the socio-ecology drivers of threat to children living within the orphan home space and its implications for nurturance care across the various complex systems of the child’s environment. The study found various factors across the complex systems of child development – microsystem, mesosystem, exosystem, microsysm and lastly, chronosystem- which undermine caregivers’ delivery and increases children’s vulnerability and risk of missing out on effective nurturance care. These vulnerabilities are endemic realities of social, and bio-ecologcal space in which child development occurs. This study recommends specialized interventions and policy directives relevant for each identified threat. It also calls for a stronger political will in improving the conditions of this category of the children while within the orphan home space and ultimately, actions towards deinstitutionalization of children.

## Introduction

Children are naturally vulnerable because of their physical and psychological levels of maturity (1). However, some children are more vulnerable due to the condition of their care and protection. The vulnerability of children is contingent on the context of their development and borders on the cumulative exposure of a child to endangering factors (2, 3). The World Bank conceives of child vulnerability in terms of the responsive capabilities of a child’s household in preventing shock, reducing the effects of shock that may arise, as well as the capacity of a household to manage the same (4). The Children’s Commissioner for England defines a vulnerable child as one at an increased risk of adverse outcomes (5). The commissioner identifies children as vulnerable when they are in state care or with any safeguarding concerns. In the same vein, the United Nations Children’s Fund (UNICEF) and the World Bank Group consider a child as vulnerable when in residential care or exposed to adverse circumstances such as extreme poverty as well as experiencing moderate-to-severe disability (6).

In Sub-Saharan Africa, an estimated 0.65 million children live in orphan homes (7). Children live within institutional care, specifically orphan homes, for a host of reasons, one of which is the loss of one or both parents (8). Generally, orphanages are institutions dedicated to housing children whose biological parents or guidance are deceased or otherwise unable or unwilling to support their children’s lives or needs (8, 9). Orphanages, therefore, house children who are in an irreversible state of abandonment or those who are there simply for care and protection until their parents are socially, physiologically, economically, or otherwise fit to cater to the care needs of their children.

Living in institutional care is highly detrimental to a child’s growth and development. Children raised within institutional care suffer structural neglect (10) and are deprived of nurturing and stimulating environments that assure them of normal growth and healthy social and psychological development. Gunnar’s three-tier classification of institutions is founded upon the quality of care provided to children and reveals that institutions are necessarily deficient by being characterized by global deprivation of the child’s health, nutrition, stimulation, and relationship needs. In some cases, where institutions provide good health and adequate nutrition support, the children are often deprived of necessary stimulation and relationship needs. The last classification is institutions that meet all needs except for stable, long-term relationships with consistent caregivers (11). Building on these, Van IJzendoorn and colleagues logically added the fourth tier, where an institutional environment is able to provide stable and consistent caregiving; however, the children are only deprived of a regular family life that is characteristic of a regular social environment (10).

Across the globe, children living in orphan homes are at high risk of poor developmental growth (12, 13). They are known to suffer diversely owing to issues relating to unfavorable/unstable staffing conditions and poor physical resources and are vulnerable to the instability of caregiving and the paucity of human interactions and attachment that is required for the development of their human capacities (14, 15). This situation produces negative consequences such as retarded physical growth of children in residential care (16), delayed cognitive performance of children (17), and difficulty in securing attachment, as well as diverse internalizing and externalizing behaviors (18–20).

Global movements driven by children’s rights to “survive, thrive, and be transformed” have called for interventions for the millions of vulnerable children who are at risk of not achieving their developmental potential. In response to this, the World Health Organization (WHO), UNICEF, and the World Bank Group launched the nurturing care framework (21) for early childhood development (ECD). Nurturing care is an intervention for many children who are at risk of not reaching their developmental potential. This practice is central to early childhood development. It creates conditions that enable communities and caregivers to ensure children’s good health and nutrition, mental development, and protection from threats. The idea, philosophy, and practice of nurturing care have continued to gain traction as an important take-off point in garnering multi-sectoral collaborations for realizing sound early childhood development (22). The practice is premised on five domains of care: promotion of a child’s health, nutrition, security and safety, responsive caregiving, and opportunities for early learning (21). The nurturing care framework rests on universal health coverage and emphasizes the important place that all sectors occupy in the promotion of sound development of children.

However, despite the growing recognition of the role that investing in early childhood development plays in realizing future global transformations and charting a more sustainable path, some categories of children may be left behind. This is more concerning given that at the heart of the Sustainable Development Goals (SDGs) is the vision to “leave no one behind,” specifically to see that all children survive, thrive, and transform.

In the provision of nurturing care, the home environment is foundational and holds a significant place in its successful delivery (23). Hence, for children who are out of their biological home setting, the organized alternate care system becomes the physical environment of importance for the realization of optimal child nurturance. African children are disproportionately affected by this adversity. An estimated 83% of countries where more than 60% of children are at risk of missing the developmental milestone are located in Africa (24).

Previous studies have identified poor parental care at conception, genetic conditions of biological parents, infections (e.g., HIV), poor child spacing, low maternal education, and parental violence as correlates of poor child nurturance care (25, 26). These factors undermine the family’s capacity to provide nurturing care for their children. However, these factors operate within family settings. They do not explain the unique experiences of vulnerability among children in orphan homes. Previous orphanage studies on child vulnerability have reported on threats such as child neglect (27), child malnutrition (28, 29), depression, and anxiety disorder (30) as common with children living within orphan homes. These studies, however, do not explain the social and ecological factors that characterize these systems and how they interact to shape adverse child development outcomes. Hence, there is a paucity of studies that focus on how the social ecology of the orphan home space contributes to child vulnerability and poor nurturance care. Identifying these socio-ecological factors is important for developing interventions to reduce the risk of poor child nurturance and strengthening the services that children receive. The present study fills these gaps in knowledge by identifying diverse aspects of child vulnerabilities within the different scopes of the child’s social ecology.

### Conceptual framework

Bronfenbrenner’s ecological system framework and the nurturing care framework were adopted as conceptual frameworks for the study. The ecological framework posits that child development occurs within a system of relationships that form a child’s environment. Bronfenbrenner argued that the environment includes five different layers: microsystem, mesosystem, exosystem, macrosystem, and chronosystem (31). The microsystem is the closest unit to the child. It includes the home environment, school, healthcare system, and other institutions that have direct contact with the child. In the context of the orphan home system, the home environment and the immediate community in which a home is situated serve as the microsystems. Hence, happenings within these close units could undermine or advance child development. In the context of orphan homes, issues relating to the social architecture of orphan homes and community members’ ill-perception of children living within orphan homes adversely interfere with child nurturance care. The mesosystem explains the dynamic relationship between the structures of the child’s microsystem. Within the orphan home system, the relationship between the representations of government (e.g., the ministry, police, court, and so on) and orphan home management) provides a suitable insight into the mesosystem. Examples of this include government orphanages’ conflicting role expectations, caregivers’ alienation by government officials, ecology of distrust, and so on. The exosystem represents the larger social unit in which, though children do not actively participate, they are greatly affected due to the considerable indirect influence it has on their interactions with any of the elements in the microsystem in which they are situated, for instance, caregivers’ mental health, work stress, role conflict, job satisfaction, and so on. The macrosystem includes the cultural- and policy-related situations in which child development occurs. The chronosystem speaks to time constructs of events that are capable of affecting child development. It accounts for the fundamentality of time in a child’s life and the major events occurring that are capable of explaining a child’s development. For instance, a child’s age has been found to inform chances for adoption, which should translate to a child’s placement within a home environment and, ultimately, safety and security. Adopting the ecological framework allows an in-depth exploration into prevailing natural and situational child ecological circumstances that foster child vulnerability.

The nurturing care framework, on the other hand, presents an eclectic intervention across different aspects of child development needs. It offers opportunities for children to maximize their potential by creating an environment where they are responsively cared for, well-nourished, have opportunities for mental stimulation, and are protected from violence and diseases (32). Nurturing care comprises five interdependent components upon which its expected outcomes are built. These are good health, adequate nutrition, safety and security, responsive caregiving, and opportunities for early learning and stimulation. The nurturing care framework enables an understanding of child vulnerabilities within the different components of nurturance care needs. Data that emerge from this study report on four major components of nurturing care (good health, responsive caregiving, stimulation/opportunity for early learning, and safety and security). On good health, the child’s mental and physical health were reported. These covered issues relating to affordability and access to healthcare delivery for children, as well as everyday events within the orphan home space that produce adverse effects on a child’s mental health, e.g., adoption of peer. On responsive caregiving, issues within this domain relate to timely responses to the child’s cues, care needs, and prompt and appropriate responses to the child’s signals. On this, caregivers’ role conflict and unhealthy mental state arising from work conditions or job dissatisfaction and care workers’ alienation likely affect responsive caregiving. Opportunity for early learning refers to having an early opportunity to relate to things, people, and objects around them for their minds to be stimulated. Here, the child’s age at entry might affect swift resilience from earlier trauma, which is capable of impairing children’s minds, placement in age-inappropriate classes, and so on. Finally, with regard to safety and security, issues relating to cultural and state policy effects on child adoption, and ill-attitudes of community members to children living within orphan homes, *inter alia*, explain the interactions between and within systems and adverse effects on procuring the safety and security of the children living within the orphan home. Taken together, these frameworks explain how happenings within and between different individual care actors and institutions that make up a child’s ecology define a child’s vulnerability, specifically in relation to the reality of procuring effective nurturance care.

## Methods

### Research design

The study used a qualitative research approach to gain an in-depth understanding of the vulnerabilities of children living within Nigerian orphan homes. Data were gathered with the use of semi-structured, in-depth interviews with orphanage managers, caregivers, and social workers. Thematic analysis was adopted to analyze the data.

### Study setting

The study was conducted in Lagos, Nigeria. Lagos state was chosen given its relatively more organized structure of orphanages in the southwestern region. The state has over 70 registered orphanages and three government-owned orphanages. Matters regarding orphanages are regulated by the State Ministry for Youth and Social Development (hereafter referred to as “The Ministry or “the State Ministry”). Orphan home is used to describe “any residence or home maintained for the reception, protection, care and bringing-up of more than six children apart from their parents but does not include any school of industries or reform schools” (33).

### Samples and sampling procedure

The sampling technique for this study was purposive. At the outset of the study, approval was sought from the Ministry for data collection. Orphanages were included based on (i) the type of ownership, whether it was private- or government-owned and (ii.) the perceived level of establishment/development. In all, 12 orphanages, including 10 private orphanages (five adjudged by the Ministry as already well-established, and five that are considered as just developing), alongside two of the three government-owned orphanages in the state, participated in the data gathering. In all, 17 respondents were engaged, comprising orphanage managers/owners (OM), social workers (SW), and caregivers (CG) in orphanages located in Lagos, Nigeria. Interview sessions lasted an average of 50 min.

### Data collection

Data were gathered with the aid of an interview guide, which was informed by the nurturing care framework developed by the WHO, UNICEF, and World Bank Group (21). The interview guide covered all components of the nurturing care framework. Items on this interview guide included the following, among others: Could you please share with me your experiences in terms of ensuring child’s safety and security within orphan homes? What roles do the communities play in promoting safety and security, good health, adequate nutrition, and responsive caregiving (nurturance care) of children? What is your take on the roles of government policies, rules, and regulations in achieving (mentioned the different components) nurturance care of children? What has been a major support to you in driving (mention the different components) nurturance care of children? Would you like to share any concern, personal or system-wise, that affects your care delivery? What major issues do you consider hampering effective delivery of (probed across the components) nurturance care? How observed irregularities influencing child vulnerabilities were also probed. Oral consent was given by the prospective respondents who had earlier been informed about all necessary details, including the purpose and modalities of the study. After the consent was granted, a convenient place and time for the interview were proposed by the prospective respondent. Before the commencement of the data collection, the study was approved by Bowen University Teaching Hospital Ethical Review Board, Approval no: BUTH/REC-647.

### Data management and analysis

Thematic analysis was adopted given its aptness for this study, which aimed at discovering common themes and perspectives from the respondents on the constellation of socio-ecological threats to child nurturance (34, 35). The analysis of the data started with the transcription of interview records to generate transcripts. The process of data analysis was informed by the ideas of Granehein and Lundman (36). First, transcripts were read repeatedly to gain a clear understanding of the contents. This process of familiarization was followed by the identification of meaning units. Meaning units are a constellation of words or sentences having an intersection of meaning or aspects relating to one another in content or context. After this, a condensation of meaning units was done. Following this, data were collapsed into themes and sub-themes. Specific themes that relate to different ecological systems were first identified, followed by the sub-systems, and finally, common categories in meaning were grouped into themes. These groupings were based on happenings within each system, for instance, microsystem feature subthemes that relate issues to the child’s microenvironment, such as homes and the community in which these homes were situated. Most suitable data were selected and quoted verbatim to support the findings. These quotes were labeled to differentiate between and within the categories of respondents. A caregiver was presented as CG, orphanage manager as OM, and social worker as SW. An orphanage manager was either the owner or one employed to oversee the affairs of the orphanage, while a social worker was either one employed by a private orphanage or a civil servant working with the Ministry. Numbers were also assigned to differentiate within the categories of respondents. For example, “SW1” means social worker 1 and “CG2” means caregiver 2.

### Rigor and trustworthiness of the study

The rigor was achieved through a number of procedures, such as member checking. This was done during data collection by intermittently summarizing the respondent submission and confirming from the respondents if it captured their opinions. This was done to ensure the intent and original contents of the respondent’s views were captured and preserved. Rigor was also achieved through reflexivity and prolonged contact with the respondents to aid understanding. Reflexivity was also deployed through the inclusion of emerging themes that were not originally captured in the interview guide for subsequent interviews.

Credibility or trustworthiness was achieved first by recruiting relevant research participants who are on the frontline of procuring nurturing care of children in orphan homes. Saturation was reached at the 10th orphan home. The credibility of the study was also established by choosing suitable meaning units that ensured no exclusion of important data as well as no inclusion of irrelevant data. For instance, under the microsystem, the social architecture of orphan homes was a suitable meaning unit that accommodates issues relating to the symbolic spiritual significance of orphan homes as well as the issues with porosity and negative external influences, while the “home-inherent emotional trauma” meaning unit was suitable for trauma arising from sudden removal/adoption of a child’s close peer, and other forms of trauma such as arising from preferential charity attention or when they realize their selves as orphans. Finally, the trustworthiness of the study was assured by presenting the findings with appropriate quotations that allow the readers to give possible alternate interpretations (36).

## Results

Participants’ characteristics table.

**Table 1:** 

SN	Label	Role	Gender	Home type by ownership
1	OM1	Orphanage manager	Female	Private
2.	OM2	Patron	Male	Private
3.	OM3	Orphanage owner	Female	Private
4.	OM4	Orphanage owner	Female	Private
5.	OM5	Matron/nurse	Female	Private
6	OM6	Matron	Female	Private
7.	CG1	Caregiver	Female	Private
8.	CG2	Caregiver	Female	Private
9.	CG3	Caregiver	Female	Government
10.	CG4	Caregiver	Female	Government
11	CG5	Caregiver	Female	Private
12.	SW1	Social worker	Female	Private
13.	SW2	Social worker	Female	Government
14.	SW3	Social worker	Male	Government
15	SW4	Social worker/nurse	Female	Government
16	SW5	Social worker	Female	Private
17.	SW6	Social worker	Male	Private

This section presents the study findings. Here, diverse aspects of child vulnerability are presented using the ecological system model. The implications of the different aspects of vulnerabilities were drawn regarding the nurturing care framework. Given that researchers have responsibilities to safeguard and protect these children, based on the study inferences, the researchers are in constant touch with the home in the form of seminars to relate the findings and spot areas where focused attention is required to improve child care delivery.

### Microsystem

The microsystem refers to institutional (i.e., orphan home-related) threats to effective nurturance care of children. It also covers children’s vulnerable experiences within their immediate communities. The social architecture of orphan homes exposes children to health threats and negative external influences. Sources of vulnerabilities within this setting include the inherent nature of care, which is generalized, conditional, and commercialized. Observed vulnerabilities at the community-level subsystem include ill-perception of orphan homes and children living within. These are discussed in the following in greater detail.

### Social architecture of institutional homes as a threat to child’s safety and health

On the social architecture of orphan homes, the symbolic significance of orphanages as a social and spiritual space attracts a wide range of individuals and groups to the home, creating situations of child vulnerability to health hazards. Several care actors mentioned this. For instance, an orphanage manager identified a home-related risk that threatens children’s health:

…you know visitors come here and they want to play with the children, some, forgetting that they have communicable diseases… some do not even know, and they still come in contact with the children. OM4/Female/Private.

Moreover, by social design, orphan homes expose children to negative external influences that make them vulnerable to ill behaviors. Orphanages are socially porous, given the way they attract different categories of people with all kinds of appearances and attitudes, leaving some unpleasant impressions on the minds of the children:

Things should be stable, but they cannot be stable because this is an open place where different people come in and go out, and different people with different characters come in contact with these children, and this is a challenge CG1/Female/ Private

…Also, the way visitors dress can influence the children… Immediately such visitors leave, we tell the children that whoever dresses this way or does one kind of hair or tattoo is not right SW6/Male/Private.

Some respondents noted that sources of negative influences included grown-up children who had developed anti-social behaviors before entering the orphan home and adults who worked within the home. A social worker narrated a story:

We used to give our children snacks to go with to school; there was this grown-up girl who used to collect the cakes from other children to sell…Last month, I declined to hold custody of a 13-year-old because we don’t want a situation whereby, they will be coming to teach those children what they are not supposed to learn. SW6/Male/Private

#### Generalized and commercialized care within orphan homes: threats to responsive caregiving

Vulnerabilities of children may also be home-induced owing to generalized care, with its resultant poor one-to-one parent–child interaction, as well as commercialized care, which characterizes orphan homes. This generally has adverse effects on a child’s behavior and responsive caregiving. Some of which can be picked from the quotes:

There is a lack of personal one on one mother–child interaction. Although we do try our best here, however, there is something missing when the child does not have that biological family setting. Psychologically, it affects their behavior. This could make some children very hard to deal with. Every person that comes, the children feel like, maybe this is their mother, maybe that is their mother. They experience attachment disorder OM4/Female/Private.

If they stay longer in the home, it will affect them. And there is nothing you can compare with one-on-one care, you know, here, we give generalized care. But at a regular home, the child will know that this is my mummy SW5/Female/ Private.

The need for primary caregivers necessitates the commercialization of care. However, this is potentially threatening to the safe and sound development of these children. For instance, commercialization hampers good care by making care conditional. Commercialized care provides a pathway for individuals who lack expertise or organic interest in child care to opt for such a sensitive job when pressured by the need for economic reward. One of a few respondents who identified with this view submitted:

You know that sometimes, people who are employed in this kind of place just want to work because there is no job out there… Taking care of children is not like handling files, a file can fall and you pick it, once you don’t take proper care of a child, it might be difficult to rectify such a mistake CG2/Female/Private.

With regard to conditional love, children living in orphan homes are not always as fortunate as their counterparts living with their original parents; this is given the differences in the degree of attachment that characterizes both categories. Attachment figures in the lives of children are the primary caregivers that protect them from threats. However, attachment between a child and parental figures varies between a social and biological parent–child relationship. The natural bond that exists in a biological parent–child relationship is observably difficult to attain in adoptive or social relationships. An orphan owner remarked:

Sometimes, caregivers recommend to me that we return children to the ministry because they are stubborn, “transfer this one because he is a problem, we cannot cope”. I don’t listen to them; I ask that if they were the ones who gave birth to the children, will they return them OM2/Male/Private.

#### Home-inherent emotional trauma impairs the child’s good health

Home-induced vulnerabilities may also materialize in the form of preferential attention that children within the home receive. The opportunities and acts of kindness received by children are different and are likely discernable to other children who receive little attention of philanthropist interest, thus communicating that some children are somewhat preferred:

A child, say, at age two or three years, at times observes when an intending adoptive parent is interested in another child. When an intending adoptive mother is interested in a child, the child plays with the prospective mother in that period of bonding, and he or she goes back to his/her room, that child is different from others, despite the fact that they are together because he or she has somebody that is coming to check up on him or her; he or she sees that person as a parent at that moment. SW2/Female/Government.

Another popularly mentioned source of threat within the home is usually recorded when a fellow child is being given up for adoption. These subject some other children to trauma:

… The sudden removal of a child exposes the remaining children to post-traumatic disorder, and for the adopted children, there are times there is no bonding opportunities. OM2/Male/Private

The statement of a social worker from another home further buttresses this point:

When they see part of their friends leaving, they will now begin to ask questions, “When am I going?” … imagine that five children are together and then, suddenly, one of them is leaving and there is no explanation for it. They’ll feel bad SW6/Male/Private.

Narrating children’s ordeal at this time, a social worker said:

… let’s now talk of the day the adoptive parents will now come for that preferred child, and the child is released to them, those ones back there will feel bad if they don’t see that child again…See, we’ve seen a scenario whereby a child is released to the parents and some of them will just sit down and start screaming, ha! ha! “When will my mummy come”? …There are some that by the time those peers leave, psychologically, they will misbehave…you understand? But if you don’t understand them, you will feel they are possessed; they are not. They are affected…you might just see one of them that has understood what is going on will just go and sit in one corner. A child who could easily go to the toilet by herself will just stay there and poop on his/her body; she is frustrated. No one will know except you understand her. There’s one child upstairs now, most of the children who came here together have been adopted, so at times, she will just switch, at times, the caregivers will not be able to understand her…She knows this one and that one has gone, so she feels, “what am I doing here?” SW2/Female/Government.

#### Public/community perceptions of orphan homes and the spiritual significance of orphans as threats to safety, health, and mental stimulation

Microsystem analysis of vulnerabilities revealed issues at community sub-systems that border on community members’ perceptions of orphan homes and the children within them. Given the cultural belief in giving to orphanage children and the fact that the majority of orphan homes rely on philanthropists and the general public for their sustenance, children may be vulnerable to charity fatigue. Respondents reported having experiences in which donors leave some undesirable conditions of donating, such as having physical contact with the children for prayers, sometimes in a manner that conflicts with the best interest of the child. Meanwhile, in some cases, failure to meet such conditions given by prospective donors led to the withdrawal of their intended support.

Some intending donors, for selfish interest, will request that all the children be brought out to pray for them, as they believe that prayers of such children are always heard by God…OM4/Female/Private…When you don’t allow them, some will rather carry their thing and go OM6/Female/Private.

Moreover, some orphanages, as a response to the educational needs of children, established schools for the children in their custody. Given the need that orphan home children have to integrate with other children, these schools are made open to other children within the community. However, community parents respond to this sometimes in a manner denigrating the status of these children living within orphan homes. For instance, some parents reportedly questioned why their children should attend a school established for orphans:

When I started the school, I called it an orphanage school, the community members did not want to bring their children. They said that they are still alive. So, I changed the name, and they started bringing their children. OM2/Male/Private.

Another orphanage owner corroborated:

Our agent told me that when she was advertising the school to some parents, they said that they learnt the school belongs to the orphanage; orphanage school, and orphan children, abandoned children, are going to be in the school, so for that reason, they were not bringing their children. That was the first time I heard such shocking words. OM3/Female/Private.

Another orphanage manager spoke of how orphanages and the children may be conceived by some community members as a dumping ground for unwanted items, even expired foods:

You know, there are even some of them that come in with items that are almost expired and you know…it’s a lot. But we are coping, we can’t complain, we are coping OM6/Male/Private.

### Mesosystem analysis of vulnerabilities in orphan homes

Data analysis on the interactions between microsystems reveals diverse aspects of vulnerabilities and threats to child nurturance. At the mesosystem, different components of nurturance care suffer, making the child vulnerable, owing to conflicting role expectations, ecology of distrusts, government–home conflicting childcare ideologies, politics of child adoption, care workers’ alienation, and officials’ apathy. Each differently puts children in vulnerable conditions. These are discussed below in greater detail.

#### Government-orphan homes conflicting role expectations: threat to all nurturance care components

Conflicting role expectations involved financial responsibilities between the government and private orphan homes. Government agencies saw orphan homes as a non-profit organization that receives grants from donors for children’s upkeep. On the other hand, the homes, specifically the private ones, lamented over the government’s neglect of the children and institutions in the face of hardship. This was more concerning for them because children living within orphan homes are widely referred to as the government’s children. These struggles implicate issues of health procurement, requisite facilities for child development, and education, feeding, and general care needs of children. For instance, even when an abandoned child is newly found, orphanages are made to be financially responsible for all medical investigations before taking custody of the child:

…Like now, we will always need to do general check-ups for newly found babies…also, children with special needs use a lot of drugs, and that is to manage their health. That is so costly SW5/Female/Private.

We (orphanages) still pay some bills. The government needs to come up with a policy that the treatment of children from orphanages has to be free. Our Matron took a child to the hospital, and they told us, we have to pay forty thousand naira (approx. 80 dollars). What is the essence of the insurance scheme if you are asking us to pay? The homes are helping the government to reduce future problems; the children are the government’s responsibility. Government looks at us as profit-making entities…they need to fund orphanages for the betterment of t society. No home can single-handedly cater for children SW6/Male/Private.

Still on procuring the health of children, many reported on how the government’s health scheme for children does not cover critical health issues. A caregiver stated:

It hasn’t been easy at all, because, you know, even if you go to government hospitals, they will not give you drugs for the children; we have to source funds to buy their drugs …. Recently the State government decides to pay for a health scheme… however, they said severe health issues are not paid for; if the child has malaria, or maybe catarrh, simple things, that’s what they pay for SW1/Female/Private.

All of the respondents from different private homes lamented over the neglect of orphan homes by the government, yet receiving pressures from the ministry to respond to the care needs of children even when the adoption charges go to the ministry:

All these facilities they said we should have; it’s money to get them. You must have a standing social worker on board, have a bus, a sick bay… they said, “the net was torn, repair it”. One day, I asked them, “Did you give me money to do all these?” …OM3/ Female/Private.

Noting that the relationship between the government and homes over matters of the children is more parasitic than collaborative, an orphanage owner sadly noted:

… The challenge is the government’s high-handedness. Supervisory organs highly handle their services, and they don’t look into what we pass through. It’s a relationship of demand without supply, and it is a very bad one. We call it a partnership, and under the partnership, one party must not gain unlimited power OM2/Male/Private.

We have seen how weak our government is, so, I do not look at them at all, because they do not love the children. Whatever they are doing is just for the income it will generate for them. You say you are the owner of the child, but you do not provide anything. We have so many staff to pay for. The government will say, as a home, we need to buy an ambulance, we must have a big generator, they say, we must have a doctor and a trained nurse. We must have a social worker. If the children are quite much, we must have a particular number of social workers. Meanwhile, you must have to pay the social workers every month. Where is the money coming from when you are the one collecting all the adoption money? OM4/Female/Private.

The government’s neglect of orphan homes, along with the misappropriation of funds generated by these homes, affects childcare management by imposing pressures on the system. The pressure manifests to threaten effective child nurturance in diverse ways. For instance, children become vulnerable in the situation of caregivers’ rash responses, thereby resulting in the disruption of responsive caregiving. The financial pressure has the capacity to create a situation where care actors become unscrupulous to meet children’s financial needs. This orphanage owner warns:

These carefree attitudes of the government could affect the children in homes in diverse ways. The orphanage owners are frustrated and could even transfer the aggression onto the children or anybody around them. The orphanage managers may not be able to put in the best…As a government, you don’t provide any of those things, you are looking for trouble because you are telling them, “You are on your own, it is either you sink or you swim …Then, if they do that, most of the stories you hear about people selling children to make money would be far less. OM4/Female/Private.

#### Caregivers’ alienation: the disruptive influence on child’s safety, security, and responsive caregiving

Further analysis of sources of child vulnerabilities at the mesosystem and its influences on children revealed orphanages/caregivers’ alienation. Several homes spoke of being relegated to nothing in the consideration of major issues that affect the welfare of children. For instance, orphanage managers and their staff reported feelings of alienation, in which, after they had devoted themselves to care of the children, even as frontline caregivers and sole financial benefactors, they are usually not carried along by the Ministry when a major decision such as adoption is being made over a child:

… what we suffer most is the attitude of treating the home as non-existent, not caring for the kind of impacts it could have on the child. They treat us as if we don’t exist. For instance, the courts don’t like to hear from the home about the child; they will rather hear from the ministry. They treat the home callously. They don’t care the children are raised there. These children are here throughout the day and night, interacting with the people you are looking down on. The funds you get, you dispose of the home of it. You make them beg for everything… In actual fact, one of the government officials referred to the home as a warehouse in one of our meetings. He said, “Look here, you don’t have any legal right over these children, you are just a warehouse. We keep our children there, and we take them when we want”. OM4/Female/Private.

Orphanage owners mentioned the demands of raising each child as well as the bond built over time. They decried being deprived of the opportunity to contribute to major decisions such as the adoption of the child:

Do you know what it takes to raise a child to a year or two thereabout? And that child will just be picked and just go from there… Everything is purely decided by the government…. there was one they adopted, the ministry officials just came and told me that they are coming to pick up the child… just like that? … Can you count the cartons of milk you gave to the child? Can you count the cartons of diapers? Can you count the medical bills you paid? the sleepless nights, the care, when the child was ill that you were afraid and prayed for him/her to survive…OM3/Female/Private.

In addition, the frontline caregivers and orphan home managers felt alienated from the children they raised and cared for and were not being granted a hearing by the court on matters of the welfare of children in their custody. Apart from the feeling of alienation, some shared a common view that, given the politics of child adoption, children became vulnerable in cases where ministry officials have a vested interest in an intending adopter. This is considered potentially disruptive to necessary adopters checks and, ultimately, the child’s safety and security:

One major problem we face is that we are not given the opportunity to take matters to court directly; we have to take the matter through the probation officer in the Ministry. The Ministry does not know the children more than us and they want to speak for us … The probation officers that are allowed to speak are working for the Ministry. Hence, if there is personal interest in the matter, the probation officer can change the matter; serving their interest, not the interest of the child. So, where is the justice? OM2/Male/Private

Issues raised on alienation also covered the issue of post-adoption checks. Some raised concerns about how the ministry alone, and never the home, could check on the child after adoption. Meanwhile, they noted that the ministry rarely does these checks. This also raises a question of ascertaining the security of children who are given up for adoption:

They reserve the right to post-adoption checks to them and not to the orphanages; we are not involved. So, their activities are not transparent to us, in the selection, in the recovery, and these are children who did not put one dime in their care OM2/Male/Private.

#### Politics of adoption: threat to child’s safety and security

Another driver of child vulnerability relating to the mesosystem is the politics of adoption, which makes a child vulnerable to unscrupulous intending adopters. This politics creates a market that is driven by financial interests and does not consider the child’s best interest. In reality, orphan homes have a list of intending adopters with an approval letter. However, these homes often receive calls to match a child with an unfamiliar intending adopter who has not gone through the requisite procedure. A common complaint is that orphan home care actors are often unaware of what is going on at this stage. This situation robs care actors of the enthusiasm to do more for other children. An orphanage manager report:

How do they (ministry officials) take care of the interest of the child when there is bias? When there is even a gang up and a scheme to manipulate in favor of their bosses (who have a special interest in some intending adopters) rather than the consideration of the interest of the child …They do anything they like; they pick our children for adoption arbitrarily. Sometimes, you don’t even know what goes on. You don’t even know the adopters. You don’t know whether they take them for rituals, and they don’t allow you to follow it up OM2/Male/Private.

Referencing the children as the ones who suffer from these irregularities of adoption, an orphanage manager reported the diversion of adoptable children from those who have been approved to another person of interest to the adoption officials. This reportedly happens without the knowledge of orphan home officials and discourages the interest of caregivers in further committing to the care and protection of the children:

The intending adopter that is preferred may have more knowledge of what is about to happen than you that has raised the child, and I am like, I have a list of people that have been on approval. Before you know it, they have handed the child over to the person of interest to them. When the next child comes, how would you feel? You will be a bit reluctant because it is like you are a nanny. Even nannies are paid now. That kind of treatment is just one example. So, the relationship between the home and the government is not cordial; it is very poor. Hence, when elephants fight, the grass suffers OM3/Female/Private.

#### Ecology of distrust: a bane to diverse components of nurturance care

Finally, on the mesosystem examination of children’s vulnerabilities in orphan homes, an ecology of distrust was discovered. This affected almost all the components of the child’s nurturance. Respondents mentioned the issue of distrust as a tragedy waning the necessary concerted efforts of care actors in manifold ways. Issues of distrust further complicate child vulnerability as it gives rise to donors’ conditional donation, failed efforts of caregivers to conduct post-adoption checks, and also explains the state Ministry’s antipathy toward adopters–orphanages’ rapport.

The following observation of an orphanage owner describes this ecology of distrust:

Another big tragedy that is affecting child welfare in the country and the state is distrust. The government does not trust orphanages; orphanages do not trust the government. The police don’t trust the government officials. The government official and social workers don’t trust the police. The Court Magistrates don’t trust the Ministry, Ministry doesn’t trust the police, and the police don’t trust the home…OM2/Male/Private.

Orphanages are established as platforms of immediate protection for children. Although orphanages are transitory in nature, children within need funds for survival and transformation during the period of their stay. Moreover, given its non-profit orientation, the majority of orphan homes absolutely rely on philanthropists and the general public for their sustenance. However, respondents reported cases of distrust that sometimes affect donors’ discouragement. For instance, some would request that children in the home should come out for them to see for fear of being swindled by orphanage operators. An orphanage matron noted:

Some of them (potential donors) feel that maybe the officials usually cart away whatever they bring…or maybe there are even no children in the home. OM6/Female/Private.

Based on her experience during the COVID-19, when she needed to reduce children’s contact with visitors, an orphanage manager recounts a story:

These donors have one thing in mind; they tell you ‘Bring out the children that we are donating for’. If you say, oh, this pandemic will not allow us to bring the children out, they will not agree with you because they also are afraid… nobody wants to sow into a deep sea that is swallowing their donation. OM4/Female/Private.

Still, on the issue of distrust, an orphanage manager reports an experience she considered unacceptable in the bid to perform necessary post-adoption checks, in the bid to perform necessary post-adoption checks:

The child was adopted and I have not been able to communicate with her adopters because each time I called them, immediately they hear my voice, they will switch off the phone and I do not know if they are thinking I am calling to ask for money or whatsoever. I have texted them but there has not been any reply so since then I have stopped because I have tried my best. OM1/Female/Private.

A majority of the respondents were of the opinion that state ministry officials played a significant role in breeding distrust between the orphanage homes, especially private homes, and the intending adopters. Orphanage actors recounted different situations where this happened. One noted how her concern for conducting post-adoption checks to ascertain the child’s welfare was condemned by the ministry and interpreted as a threat when an adopter who was averse to post-adoption checks accused the orphanage manager of threatening her parenthood:

They summoned me and started asking me why I was threatening somebody’s parental skills. “Do not you know when a child is taken away from your home, you do not have the right to even say anything, whether she’s okay or not? She (the adopter) is capable *OM3/Female/Private*; …Every effort you make is criminalized. *OM2/Male/Private*.

Furthermore, on the ecology of distrust, most private orphanages are not self-sustaining and rely on charity in the face of the government’s negligence. An orphanage owner complained bitterly about the government’s failure to ease their financial burden or at least disburse proceeds from adoption charges to the orphanage. Some condemned the ministry for failing to establish schemes or float policies that foster healthy support for the orphanage but rather serving as an antagonist to receiving help from the intending adopters. She expressed this concern:

The Ministry is the one collecting all the adoption money. You collect them and tell the adopters not to give us anything. They actually instruct them: “these are government’s children, once you pay the adoption charges don’t let any home tell you any story; those homes are very crafty. They want to sell the children to make money”. So, when you as an adopter hear that, you just come to the home to ask, “Where is the government child, give me and let me go”. They are not helping in any way *OM4/Female/Private*.

This ecology of distrust makes children vulnerable in countless complex ways and across the nurturing care components. This includes a lack of funds to cater to various social and healthcare needs of children.

### The exosystem and threats to effective nurturance care

The exosystem represents the larger social unit. Although a child does not actively participate in this system, they are greatly affected positively or adversely by this system due to the influence it exerts on children who interact with any of the elements in the microsystem in which a child is located. The exosystem, among other things, speaks to issues that concern the mental health and stability of caregivers.

#### Caregivers’ role conflict/dispiritedness: threat to responsive caregiving

In this study, evidence that aligns with this reveals that caregivers’ role conflict and care workers’ dispiritedness threaten effective, responsive caregiving. On this, some respondents mentioned how it might reflect workers’ characteristics such as age, experiences on the job, family characteristics, and reproductive age status. These are likely to have spillover effects on the quality-of-care delivery:

I think in an institution like this, anybody that will work here should be someone that has experience in taking care of children; not that they will bring a young girl to work here, because… they are not patient. They want to go to the beach, go here and there; the effects go to the children, but as a mother, a grandmother, you know you have to give these children all your attention, you know that anywhere you miss it, it tells on the child as they grow up. Sw4/Female/Government.

Similarly, caregivers’ conflicting roles might affect the care children receive in orphanages. A caregiver advised that older women are more suited to provide undivided attention to children in their care. In her words:

My children are grown up, so there is nothing that I will say I want to go home to do, I want to go home and cook for them, I want to go home and take care of them? They are all grown up. But in a case where you have a nurse that is still under child bearing age and newly married the attention is usually divided.CG4/Female/Government

The implication of this counsel may be seen in the quality of responsive caregiving that children receive. Similarly, caregivers’ poor family communication and lack of social support also relate to effective, responsive caregiving. Some orphan home mothers discussed how effective communication and social support within their biological home helped to foster optimal care delivery:

I usually make my children understand that just as they go to school and have a time for closing, I also have to go to work and come back when it’s my closing time. OM1/Female/Private.

An orphanage manager speaks of spousal social support as helpful in averting role conflict:

…It doesn’t in any way because my husband is very supportive. So, it’s the same way I will treat my biological children that I will treat these children OM6/Female/Private.

Still, on the exosystem, co-worker’s non-compliance might discourage those who aspire to excellence in their roles:

When you are trying to correct people working, and they are like, what is she saying? Do you understand? In your own way, because of your level of exposure…at times it’s not only them, it might even be your boss and you are trying to explain something to, and they are being adamant about it, or at times, when there are innovations, and you are trying to bring it in, and you are feeling frustrated, you know that you, yourself will be psychologically affected OM2/Male/Private.

The major source of discouragement is the nannies, you know...a lot of them come in here and they are like, “we have passion for children”, but when they get in here, it’s something else they do. Some even abuse the children emotionally…if you say you have passion for children, then, treat them like children, not that you won’t even listen to them, you punish them at will and you are not trying to understand each child with her/his own peculiarities OM6/Female/Private.

Negative attitudes toward children may be attributed to workers’ dissatisfaction. The care of children entering the orphanage calls for the services of paid primary caregivers who protect them from threats. However, the commercialization of care is potentially threatening the safe and sound development of these children. For instance, a change in the condition of services is also likely to produce dysfunctional consequences on the quality of care delivery. Respondents reported the different ways in which these manifests affect responsive care delivery. Some explained this conditional care as fostered by poor economic situations that pressured caregivers into opting for such sensitive jobs as child care where they lack the interest or expertise. An orphanage owner submitted:

You know that sometimes, people who are employed in this kind of place just want to work because there is no job out there… Taking care of children is not like handling files, a file can fall and you pick it, once you do not take proper care of a child, it might be difficult to rectify such a mistake OM2/Male/Private.

...it’s more about the money they earn… It’s this money factor. They are just out to make ends meet, not because they love these children. They seek for employment to take care of themselves. They tell you they have passion for children, but when you employ them, they do something else…If we relate with these children on the same level, then we will be bringing out well-groomed nurtured, equipped, emotionally sound children, we won’t have issues OM6/Female/Private.

The commercialized system of care produces adverse effects on child nurturance in situations of dissatisfaction of caregivers due to poor or delayed payment, absence of incentives, or in the event of organizational restructuring (e.g., downsizing). Workers’ dissatisfaction generally lends to their lack of enthusiasm on the job, ineffectiveness, or workers’ low commitment to work, however, the reality of this within a childcare domain is very concerning given the sensitivity of such a space as live-involving. A caregiver noted that her continuity on the job is driven by a lack of alternate options:

Some things that get me discouraged, but what do we do? This is due to the global economic downturn. The problem there is poor salary…The salary is too poor, and then, you will think, should I continue, should I leave the job, and then you will think if I leave, “what else will I do?” CG1/Female/Private.

… that is why at times you will come here and tell a caregiver to do this and she will be frowning at you, you will not blame the person, somebody that has not slept for the whole day, and at the end of the day, they will just think, “how much are we even collecting, how much are they giving us?... SW2/Female/Government.

Commenting further on this, a social worker identifies this problem as a pathway to ineffective nurturance care of children:

Staff welfare is very important, in terms of salary and bonuses to encourage the caregivers to work very well. If you are not happy, there’s no way you can work effectively. For instance, I’m a caregiver, and I don’t go home for a week, that sacrifice is fair enough…such a person needs to be well motivated. If you have a nanny and you don’t motivate the nanny, your child will suffer, talk less of someone that is now taking care of 4, 5, 10, 15, 20 and 25 children SW6/Male/Private.

Downsizing also means that duties may be more burdensome for those retained.

…with the poor economic situation of the country now, we have to downsize the number of staff here. That was what they did so that they will be able to pay salaries to the few ones left…SW4/Female/Government.

### The macrosystem and vulnerabilities of children living within orphan homes

The macrosystem takes account of cultural- and policy-related situations in which child development occurs. Some unfavorable religious and cultural beliefs could hamper children’s chances for effective nurturance care and the optimal development of children in orphan homes. Children living in orphan homes are sometimes vulnerable to some cultural outlooks that define and redefine their significance within cultural groups. For instance, African cultural values for women’s fertility and childbearing create a natural demand, and sometimes, in a spirited manner, a “market” for children where infertile individuals may clandestinely manage their infertility. This earns infertile women social validation even through the state system of adoption. This situation creates an opportunity for shady practices capable of jeopardizing the concerned child’s security and safety.

#### Cultural outlook to child adoption–threat to safety and security of adoptable children

Child adoption was originally designed as a life-saving option for children in an irreversible state of abandonment. However, its use as a strategy for managing infertility allows for intending adopters to choose a preferred child. This, in turn, creates a situation where some children are treated as “undesired” and are vulnerable to emotional violence. Speaking to the observed reality of cultural differences in the purpose for adoption, culturally, the majority of Nigerians opt for adoption to manage infertility rather than for altruism. A child’s age and health history chiefly inform intending adopters’ preferences. Thus, the act of adoption is culturally conditioned to meet the esthetic and functional demands of the adopters; hence, some children are not desired locally. An orphanage owner remarks:

If you are here, occupying this seat, you will see how our culture is limiting us. It is a shame… What kind of love do we have in Nigeria? I am sorry, I have seen it all here; very selfish love, conditional love. If a child does not have long hair, if you are not fair, I cannot take you… conditional love. The White people do not care. If you see those coming to seek to adopt HIV positive children, you will begin to ask yourself, am I a good human being? This job has humbled me greatly. Nigerians do not have love… they are so mechanical in their love approach. The White man that came to adopt one of our children that is reactive has already a Nigerian daughter he adopted that is deaf and dumb. He said, “Do you know I adopted one earlier that is deaf and dumb? Then, I am coming back because I want to help another.” That White man had to learn sign language because of her. How many Nigerians would come here to take a child with special needs? …Some others would turn the child around, and would complain… *Hey…E no get hair* (She is not hairy), in our family, we have plenty of hair. This one is too short… in our family we are tall… sometimes they yell at them…open your teeth! That is the kind of experience they have with Nigerian adopters, and the poor child would be looking. That is the slave trade…OM4/Female/Private.

#### State policy ill-attitude to inter-country adoption strains the opportunity for the child’s placement for care and protection

The negative attitudes of representatives of the state to inter-country adoption of children do not favor children not locally desired, with resultant effects in children’s prolonged stay in institutional care. While intercountry adoption has its risks and adversities under poor regulation, it may be a life-saving option and might provide opportunities for children to reach their full potential when they are not locally desired by intending adopters:

They should allow those children to go, Nigerians would not adopt any child with brain issues or a deaf and dumb child. These kinds of children would remain in the orphanage forever. Even the ones that are HIV positive get adopted by foreigners... I remember a child, when he came, you cannot stand his look; he had no teeth. He looks very ugly in the Nigerian way, but he’s been adopted out of the country. The adoptive parents love him so much, they will kiss him, and show love to him in different ways. These children would have been left behind. Let them go and achieve OM4/Female/Private.

### Chronosystem and child’s vulnerabilities to poor mental stimulation

The chronosystem refers to time constructs of events capable of affecting child development. It accounts for the fundamentality of time in a child’s life and the major events occurring that are capable of explaining a child’s development. On opportunity for mental stimulation, some caregivers and social workers reported how the age of entry into the orphan homes determines a child’s opportunity for resilience from earlier exposure to traumatic experiences when placed within a development-oriented and functional orphan home:

Children in the home have different backgrounds and some have not been to school before, some are children that were rescued, some are vulnerable children that came in from a very pathetic situation, and have not even passed through school or have whatsoever type of education. We’ve had children in this home that were rescued from their mentally challenged mothers, for instance, while they were with their mothers, what they did was go to parties and pick the foods from floor. For those kinds of children, before you can stabilize them, it takes a whole lot, because, for a while, after giving them food, they will still pick the floor because they are fond of it… But for a child that came in like a day old to the orphanage, those kinds of children are sometimes almost perfect because you can give them the training from the beginning SW5/Female/Private.

The child’s age of entry into an orphan home was reiterated by some orphan home managers, stating its implication on children’s early enrolment in school, class appropriateness, and ultimately, the child’s mental health in the group:

…Well, in terms of age specificity, the problem is an issue with older children who enrolled late in school, they get depressed, some were enrolled at age 11, and joined the home, say, age 8, meanwhile, they had not learnt anything before in their lives. So, when we ask them to go to school, they will have to start from kindergarten 1 or Primary 1, and they find children that are seven years older than in the same class, it affects them, and some do not cope. SW3/Male/Government.

Furthermore, the age of a child determines her or his chances of adoption. Meanwhile, the adoption of children is designed to serve to assure the growth and development of children in an irreversible state of abandonment by placing them within adoptive homes where their optimal development is assured. However, several respondents complained about the difficulty in placing children once they cross the preferred age for adoption. This is because the majority of those who now adopt instead do it to manage infertility. Some said:

Well, by the Nigerian standard, once a child can speak and know him/herself, people shy away from adopting such. Although there is some improvement these days, when they can’t get days old babies, they adopt toddlers, after much persuasion, and pleading because they want the child to be settled, but still, not all of them will go. Anyone not adopted, you have to keep within the home. OM4/Female/Private.

We have a child of 14 years here, and the child has been here since when she was 2 years, nobody wants to take her since that time, even right now, we are looking for whom to adopt her, but we have not found any SW1/Female/Private.

Children who are older than the adoptive age might face the risk of prolonged stay in orphan homes and may become more vulnerable to poor care.

### Discussion of findings

Socio-ecological drivers of vulnerabilities of children living in orphan homes cut across the various systems of relationship that form their nurturance care and development. These vulnerabilities are inherent parts of everyday realities, operations, and endemic social forces relating to orphan home space. These include care workers’ poor mental state, some state laws and policies regarding child welfare, commercialized nature of care, and social architecture of orphan homes, *inter alia*.

**Figure 1:**
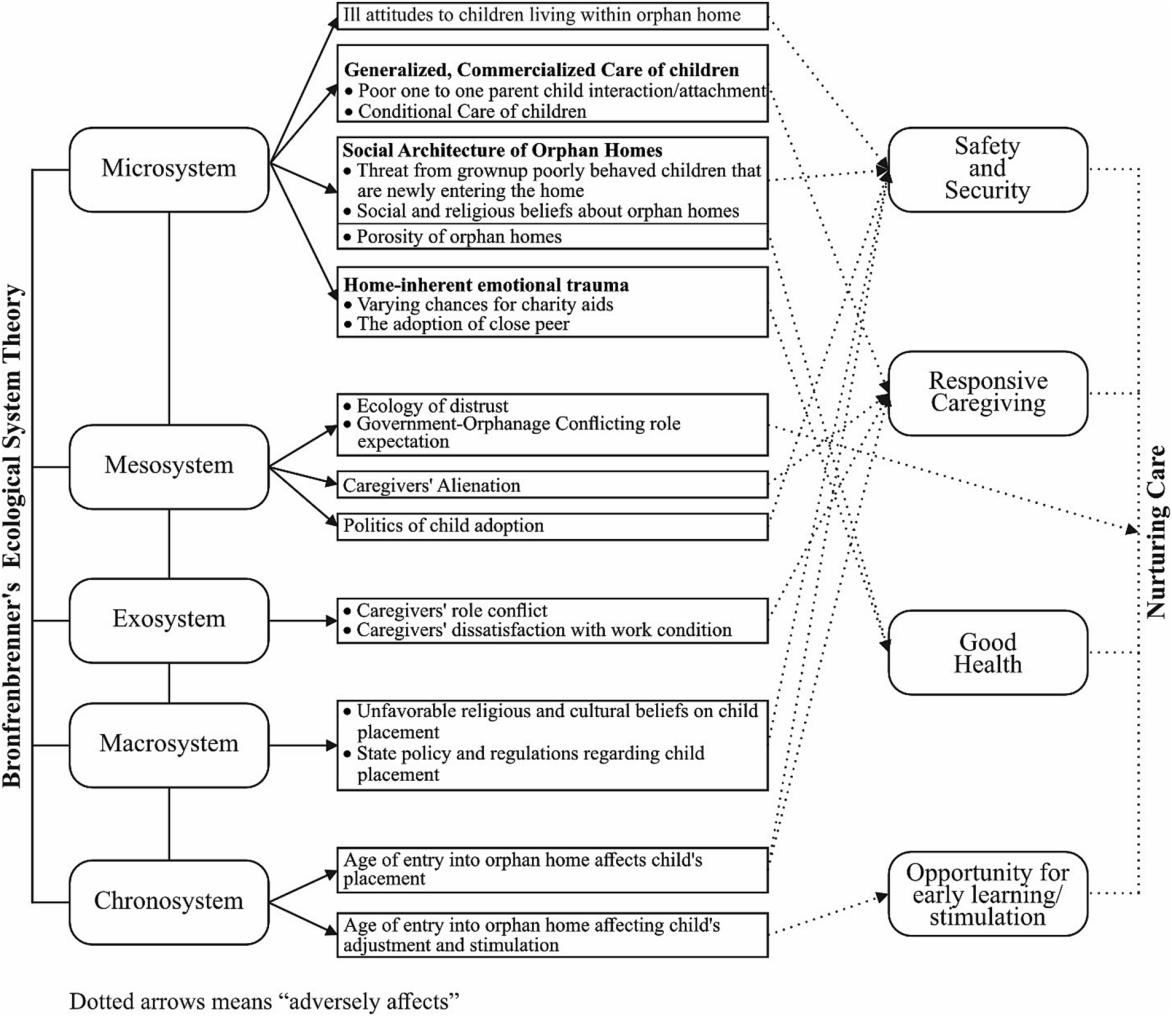
Aspects of child vulnerabilities and threats to nurturing care, using Bronfenbrenner ecology theory.

At the microsystem, we identified a number of threats to effective nurturance care. Institution-related threats included negative external influences stemming from the social architecture of orphan homes. These influences expose children to social and health risks due to visits made to this space by diverse individuals and groups. They also face threats from already grown-up children newly entering institutional care and also mental health threats from sudden removal of peers. In addition, the generalized nature of care that children receive in orphanages subjects them to some vulnerabilities and potentially threatens responsive caregiving (please see Figure 1). Observed vulnerabilities in the community sub-systems include negative perception of orphan homes and stigmatization of children living within these homes. The community’s negative perceptions were also evident in parents’ refusal to enroll children in orphanage-owned schools, leading to a feeling of stigmatization by the children. This study not only supports findings from previous works reporting feelings of stigmatization among the children (37, 38), but it also shows how this contributes to the stigmatization of the children. Previous studies (39, 40) have identified orphanage children and adolescents with emotional issues such as post-traumatic disorders. These early experiences of trauma could set the stage for the diverse emotional problems that are identified among children living within orphan homes in their later years. Although trauma arising from their identity is socially produced, it points to the ill-social construction of homes and the children within as “irregular.” Hence, regularizing these children calls for deinstitutionalization.

Moreover, ensuring care for children entering the orphanage calls for the services of paid home caregivers. However, the commercialization of child care is potentially threatening the safe and sound development of these children. This is due to the conditional care offered as a service, which is often not child-centered. For example, some caregivers are pressured into taking up these roles by poor economic conditions. Some lack the interest or expertise to function effectively. This may be further aggravated by caregivers’ dissatisfaction with work conditions. Van IJzendoorn and colleagues’ (10) findings resonate with observations from our study that situates disruptive nurturance within necessarily commercialized care of children. However, they observed this as arising from high staff turnover and caregivers’ shifts/vacations, all of which are markers of the commercialization of care. Furthermore, the attachment process and the nature of the bond that exists in a biological parent–child relationship are difficult to attain in non-biological relationships. This sets the stage for conditional care. Van IJzendoorn and colleagues (10) also observed that caregiving duties are made in a business-like manner. This supports our idea of care conditioning and commercialization of child care. This study shares similarities with that of Boadu, Osei-Tutu, and Osafo’s study (37), which also observed children’s difficulty in building an emotional bond despite caregivers’ affectionate care. This observed difficulty instructs on the urgent need for deinstitutionalization. In recognition of the ills arising from the institutionalization of children across the globe, policymakers and major international instruments and entities (41–44) concerned with the right welfare and protection of children have continued to campaign for a family-based system of alternate care as against institutionalization.

At the mesosystem, different components of nurturance care suffer, thus making the child vulnerable owing to aspects of vulnerability such as conflicting role expectations bordering on financial responsibilities toward children’s health procurement, education, and general care needs. Ecology of distrust, politics of child adoption, care workers’ alienation, and officials’ apathy were also identified with this component. The idea of nurturing care provides a suitable space for multi-sectoral collaborations for early childhood development (22). However, distrust undermines the concerted efforts of care actors. For example, such strained cordiality was reportedly intended by the ministry to forestall situations where orphanages place undue financial demands on prospective adopters. However, this functioned to obstruct opportunities for donation with resultant effects on all components of nurturing care. In addition, the strained cordiality arising from distrust poses an obstruction to post-adoption-check conduct by the orphanages, which threatened the security and safety of children that were to be stabilized by adoption.

At the exosystem, children were found to be vulnerable to caregivers’ role conflicts (conflicting duty calls within caregivers’ biological home, workplace, and orphan home), care worker’s dispiritedness, which was often a reflection of workers’ lack of experience, and some caregivers’ characteristics such as poor quality of family life of caregivers. However, effective communication and social support within their biological homes were reported to foster optimal care of children in their custody. However, engaging young folks with no childrearing experience was identified as a disadvantage to child nurturance caregiving as inexperienced young folks were often unable to give the required attention to children. Our findings agree with Bettmann, Mortensen, and Akuoko’s view that the vulnerabilities of children in orphan homes are situated within the lack of requisite knowledge of caregivers in understanding children’s emotional needs (45). This study, however, takes a further step to situate caregivers’ knowledge deficit in lack of experience and an absence of organic interest in child caregiving roles. In terms of best practices, engaging older women who would give undivided attention to orphan home children is worth considering.

At the macrosystem, religious and cultural beliefs hampered children’s chances for optimal development in complex ways. Children living in orphan homes are often vulnerable to some cultural outlooks that define and redefine their significance. African cultural values for women’s fertility and childbearing create a market for children where infertile individuals may clandestinely manage their infertility pressures, sometimes illegally, in a matter that poses a threat to children’s safety and security through different shady practices that disrupt the opportunity to observe due process (46–48). This threat within the system of adoption has been connected to the observed gap between the high demand for children and the low number of adoptable children within the system, creating an opportunity for shady practices that threaten adoptable children’s security and safety (13). Specifically, the spirited competition that results from this shortage potentially threatens the philosophy of altruism and humanitarianism that define orphanages by replacing it with infertility management (49). This shifts adoptable children away from the center of adoption practice in a manner that is capable of jeopardizing children’s safety and security. Meanwhile, international and local instruments, for instance, the CRC, Art. 21 (50), and Hague Convention, Art. 19a (44), emphasize the consideration of the best interest of the child as paramount in all matters, including adoption-affecting children.

At the chronosystem, especially in regard to the opportunity for mental stimulation, this study observed that the age of entry into the orphan homes determines a child’s chances of developing resilience against traumatic experiences. Similarly, it was observed that children are difficult to place in foster homes once they cross the preferred age for adoption. These children experience prolonged stays within the institutional homes, which increases their vulnerability to developmental threats.

Finally, this study has identified several factors within the ecology of child care and development that have the potential to interfere with the chances of effective nurturance. Given that most of these aspects of vulnerabilities were characteristics of their living condition and policies regarding their care, this study calls for political actions toward the deinstitutionalization of children. It also makes a case for responding to various home-induced vulnerabilities of children. Finally, it calls for specialized training and retraining of child care actors on managing the unique experiences of the children.

## Data availability statement

The original contributions presented in the study are included in the article/supplementary material, further inquiries can be directed to the corresponding author.

## Ethics statement

The studies involving humans were approved by Bowen University Teaching Hospital Ethics Committee. The studies were conducted in accordance with the local legislation and institutional requirements. The participants provided their written informed consent to participate in this study.

## Author contributions

OO conceived the research, drafted the instrument, collected the data and analyzed and did the report writing. GH edited through the stages. All authors contributed to the article and approved the submitted version.
